# DNA microarray chip assay in new use: early diagnostic value in cutaneous mycobacterial infection

**DOI:** 10.3389/fcimb.2023.1183078

**Published:** 2023-07-01

**Authors:** Qian Yu, Yuanyuan Wang, Zhiqin Gao, Hong Yang, Siyu Liu, Jingwen Tan, Lianjuan Yang

**Affiliations:** Department of Medical Mycology, Shanghai Skin Disease Hospital, Tongji University School of Medicine, Shanghai, China

**Keywords:** DNA microarray chip, early diagnosis, cutaneous mycobacterial infection, nontuberculous mycobacteria, skin tissue culture

## Abstract

**Introduction:**

The clinical practicability of DNA microarray chip in detecting the presence of mycobacterial species/isolates directly in the skin tissues has not been evaluated, nor the efficacy of DNA microarray chip as a novel diagnostic tool for the early diagnosis of cutaneous mycobacterial infections is known.

**Methods:**

The present study analyzed the incidence of cutaneous mycobacterial infections in Shanghai and explored the efficacy of a novel DNA microarray chip assay for the clinical diagnosis of the disease from skin tissue specimens compared to traditional detection methods. A total of 60 participants fulfilling the defined diagnostic criteria and confirmed positive for cutaneous mycobacterial infections from 2019 to 2021 were enrolled in the study. Subsequent to recording the participants’ medical history and clinical characteristics, the skin tissue specimens were collected for analyses. The specimens underwent histopathological analyses, skin tissue culture, and DNA microarray chip assay.

**Results:**

Increased incidence of cutaneous mycobacterial infection was detected from 2019 to 2021. The most common infecting pathogen was *M. marinum* followed by *M. abscessus*. The sensitivity, specificity and accuracy of the skin tissue culture method were 70%, 100% and 76.62%, respectively, while that of the DNA microarray chip assay were 91.67%, 100% and 93.51%, respectively. The sensitivity and accuracy of the DNA microarray chip assay were significantly higher than those of the skin tissue culture method. The positive likelihood and diagnostic odds ratio were >10 and >1, respectively for both the methods. The negative likelihood ratio was significantly higher (30% vs 8.33%) and the Youden’s index was significantly lower (70.00% vs 91.67%) in the skin culture method compared to that of the DNA microarray chip assay. There was a significant association of false negative results with a history of antibiotic use in the skin tissue culture method.

**Discussion:**

Given the increasing incidence of cutaneous mycobacterial infections, early diagnosis remains a prime clinical focus. The DNA microarray chip assay provides a simple, rapid, high-throughput, and reliable method for the diagnosis of cutaneous mycobacterial infections with potential for clinical application.

## 1 Introduction

Cutaneous mycobacterial infection is an infectious granuloma of the skin and subcutaneous soft tissue caused by pathogens commonly found in clinical settings, *Nontuberculous mycobacteria* (NTM) and *Mycobacterium tuberculosis* (MTB) (Gardini et al., 2022). The incidence of cutaneous mycobacterial infections has been steadily increasing over the past 10 years, with a higher rate of occurrence due to factors such as the rising prevalence of HIV infection, increased use of immunosuppressants, growing geriatric population, and cosmetic or surgical procedures (Mei et al., 2019). This group of diseases are frequently underdiagnosed and delayed treatment results in significant stigma, disfigurement, deformity, and disability.

Diagnosis of cutaneous mycobacterial infection is challenging because of the wide spectrum of nonspecific clinical manifestations and histopathological similarities including the granulomatous reaction pattern (Min et al., 2012; Franco-Paredes et al., 2018). Conventional culture methods are considered the ‘gold standard,’ but they are often time-consuming and have a lower positivity rate, resulting in delayed patient care (Kromer et al., 2019). Recently, several studies have reported the use of various molecular diagnostic techniques, such as gene sequencing, PCR analysis, and high-performance liquid chromatography, that could used for the rapid identification of mycobacterial species/isolates (Kim et al., 2015; Kim et al., 2018; Shen et al., 2020). These have been gradually accepted by dermatologists as an auxiliary in the diagnosis of cutaneous mycobacterial infections. However, the clinical application of these techniques is limited due to their being labor-intensive, less efficient, and expensive. Thus, development of a rapid, simple, high-throughput, and accurate diagnostic method for the detection of mycobacterial species/isolates remains the need of the hour.

DNA microarray chip technology has emerged as a promising tool for the rapid detection of mycobacterial species (Tobler et al., 2006; Gaballah et al., 2022). Zhu et al. established a DNA microarray platform based on the principle of polymorphism in the 16S rRNA space region for multi-target, rapid, and simultaneous detection of 17 pathogenic mycobacteria associated with pulmonary mycobacterial infection, including NTM and MTB (Zhu et al., 2010; Shi et al., 2012). As a diagnostic tool for pulmonary mycobacterial infections, DNA microarray chip technology enables high throughput and rapid detection of mycobacterial isolates in sputum specimens. However, their widespread application for the clinical diagnosis of dermatological diseases, still needs to be explored.

This retrospective study aimed to investigate the incidence and clinical characteristics of cutaneous mycobacterial infections, explore the clinical practicability of DNA microarray chip in detecting the presence of mycobacterial species/isolates directly in the skin tissues, and performs a comparative study with traditional methods to analyze the efficacy of DNA microarray chip as a novel diagnostic tool for the early diagnosis of cutaneous mycobacterial infections.

## 2 Materials and methods

### 2.1 Clinical sample collection

The present retrospective study was conducted at the Shanghai Dermatology Hospital, China. Patients admitted to the hospital from January 2019 to December 2021, who were suspected to be positive for cutaneous mycobacterial infection were included in the study. The study was reviewed and approved by the Ethics Committee of Shanghai Dermatology Hospital. The participants provided their written informed consent to participate in the study and for the publication of any potentially identifiable images or data included in this article. Details of participants, including age, sex, signs and symptoms of disease, medical history, contributing factors, therapeutic schedule, and prognosis were documented. Clinical samples were taken from participants and evaluated for hematology and blood chemistry, skin biopsy, skin tissue culture, identification of microbial isolates, and DNA microarray chip assay. Skin tissue specimens from participants were divided into three parts (6×6×6 mm, each) and processed for histopathological analyses, tissue culture and pathogen identification, and DNA microarray chip assay. Contaminated skin tissue specimens that were extremely small were excluded from the study.

Control group: The 17 mycobacterial reference strains were used as positive controls. The negative controls were skin samples from 8 patients who were diagnosed with deep dermatophytosis, 5 with pigmented nevus, 2 with epidermoid cysts and 2 with seborrheic keratosis.

### 2.2 Culture and gene sequencing

Skin tissues were excised, ground, and decontaminated with N-acetyl-L-cysteine-2% sodium hydroxide (pH 6.8) according to the standard protocol. Bacteria suspended in phosphate buffered saline (PBS) were concentrated by centrifugation at 3000 x *g* for 15 min and the sediment was resuspended in 0.5 mL of sterile water for use as skin tissue homogenates for standby application. Further, skin tissue homogenates were cultured in Lowenstein-Jensen media at 30 °C and 37 °C in 5% CO_2_ and in Sabouraud dextrose agar (SDA) at 26 °C and 35 °C. Although NTM species grow within 2-3 weeks on subculturing, the cultures in the present study were incubated for 6 weeks. The suspected samples with *M. ulcerans* or *M. genavense* were stored for 8-12 weeks. Cutaneous MTB cultures were cultured for 8-12 weeks. The cultured organisms were characterized as acid-fast bacilli by Ziehl-Neelsen staining. Subsequently, mycobacterial genomic DNA was extracted, 16S rRNA gene was amplified by polymerase chain reaction (PCR), sequenced, and the obtained sequences were compared using BLAST for the identification of mycobacterial species.

### 2.3 DNA microarray biochip assay

The DNA microarray biochip (CapitalBio Company Ltd, Beijing, China) used in the study could identify 17 mycobacterial species, including *M. tuberculosis*, *M. intracellulare*, *M. avium*, *M. gordonae*, *M. kansasii*, *M. fortuitum*, *M. scrofulaceum*, *M. gilvum*, *M. terrae*, *M. chelonae/M. abscessus*, *M. phlei*, *M. nonchromogenicum*, *M. marinum/M. ulcerans*, *M. aurum*, *M. szulgai/M. malmoense*, *M. xenopi*, and *M. smegmatis*. The DNA microarray biochip test was performed as described previously (Zhu et al., 2010). Firstly, skin tissue homogenates were processed for DNA extraction and target gene fragments were amplified by PCR using 16S rRNA. PCR was performed in two amplification rounds, with an initial activation step at 37 °C for 10 min, denaturation at 94 °C for 10 min, followed by 35 cycles of first round exponential amplification at 94 °C for 30 s, 60 °C for 30 s, and 72 °C for 40 s, followed by 10 cycles of second round linear amplification at 94 °C for 30 s and 72 °C for 60 s, and final extension at 72 °C for 5 min. Secondly, the amplified and fluorescently labeled gene fragments were hybridized with probes on the DNA microarray chip. Probes represented species-specific 16S rRNA gene sequences for the identification of different Mycobacterial species. Thirdly, the chip images were analyzed using a LuxScan Dx24 microarray scanner (CapitalBio Company Ltd, Beijing, China) and the fluorescent intensities were quantified by the mycobacteria identification array test system software (CapitalBio Company Ltd, Beijing, China).

### 2.4 Diagnostic criteria

In the present study, diagnosis of cutaneous mycobacterial infection was based on the combination of clinical and microbiological evidence. A participant was defined as positive for cutaneous mycobacterial infection, if they met all of the following diagnostic criteria: suspected of having cutaneous mycobacterial infection; skin biopsy indicated infectious granuloma irrespective of the Ziehl-Neelsen staining; one or more positive result confirming mycobacterial infection with any of the microbiological detection methods used, including skin tissue culture and DNA microarray chip or macrogenomic detection of pathogenic microorganisms; and participant cured after combined anti-mycobacterial therapy.

### 2.5 Statistical analysis

Data were analyzed using the SPSS version 20.0 software (Armonk, IBM Corp., NY). The median and interquartile range (IQR) was calculated using descriptive statistics. Sensitivity, specificity, and accuracy of clinical diagnosis between two categories of samples were calculated and compared. The effectiveness of the methods used to diagnose cutaneous mycobacterial infection was compared using the likelihood ratio, the Youden’s index, and the diagnostic odds ratio. The sensitivity, accuracy, negative likelihood ratios, and Youden’s indices were compared between the groups using Chisquare tests. For the skin tissue culture method, risk factors associated with false-negative results were identified using logistic regression analysis. All statistical tests were two-sided, and results were considered significant at a *P* < 0.05.

## 3 Results

### 3.1 Predominance of NTM over MTB infections in cutaneous mycobacterial infected participants

A total of 77 suspected cutaneous mycobacterial infection cases were enrolled for the study, of which 60 cases were confirmed with cutaneous mycobacterial infection as they met the specified diagnostic criteria, 12 cases were diagnosed with cutaneous deep dermatophytosis through skin tissue culture, and 5 cases were diagnosed with non-infectious granuloma by skin biopsy. The number of cutaneous mycobacterial infection cases was in general increased during the study period (**Supplementary Figure 1**). Cutaneous NTM infections were more prominent than MTB infections in the study population (91.67% and 8.33%, respectively). The most common NTM species detected were *M. marinum* (58.33%), followed by *M. abscessus* (30.00%), and *M. chelonae* (3.33%), respectively (**Table 1**).

**Table 1:** Profile of mycobacterial species detected in participants with cutaneous mycobacterial infection (n = 60).

	Year of diagnosis, No. (%) of isolates			
	2019 (n = 8)	2020 (n = 16)	2021 (n = 36)	Total (n = 60)
Mycobacterium species	n (%)	n (%)	n (%)	n (%)
***M. tuberculosis***	1 (12.50)	2 (12.50)	2 (5.56)	5 (8.33)
**NTM**				
***M. marinum***	6 (75.00)	10 (62.50)	19 (52.78)	35 (58.33)
***M. abscessus***	1 (12.50)	3 (18.75)	14 (38.89)	18 (30.00)
***M. chelonae***	0	1 (6.25)	1 (2.78)	2 (3.33)

### 3.2 Clinical features of participants with cutaneous mycobacterial infections

Clinical features and causative factors of cutaneous mycobacterial infection of participants are summarized in **Table 2**. The infected male-to female ratio was 1:2.75, indicating a selective predominance of infection in females. The median age of participants was 52.5 years (range: 10-82 years). The time lapse between the onset and diagnosis ranged from 1 month to 40 years. The median course of cutaneous mycobacterial infection was 4 months (range: 1-480 months) and the causative factors were recorded in 53 participants. Aquatic trauma was reported by 24 participants (40.00%, 23 cases of *M. marinum* and a case of *M. chelonae* infection), while non-aquagenic trauma was found in 7 cases (11.67%, 5 cases of *M. marinum* and 2 cases of MTB infection). Cosmetic procedure preceded infection in 19 participants (31.67%, 18 cases of *M. abscessus* and a case of *M. chelonae* infection). BCG vaccination preceded one case of MTB infection (1.67%), while tuberculosis was associated with 2 cases of MTB infection (3.33%). Several participants had a history of antibiotic use before consultation, either in the form of systemic administration or tropical ointment (23 cases (38.33%) and 15 cases (25.00%), respectively.

**Table 2:** Clinical features of participants with cutaneous mycobacterial infection (n = 60).

Characteristics	n	%
Sex		
Male	16	26.67
Female	44	73.33
Age		
0~39 years	23	38.33
40~59 years	21	35
≥60 years	16	26.67
Median (range)	52.5 (10~82)	
Duration, mo.		
≤3	26	43.33
>3,≤6	15	25
>6	19	31.67
Median (range)	4 (1~480)	
Causative factor		
Aquatic trauma	24	40
Nonaquatic Trauma	7	11.67
Cosmetic procedures	19	31.67
Iatrogenic procedures	1	1.67
Internal focus	2	3.33
None	7	11.67
History of antibiotic usage		
Systemic antibiotic	23	38.33
Topical antibiotic	15	25
None	22	36.67
Initial location of skin lesion		
Hand	33	55
Face	18	30
Forearm	3	5
Neck	2	3.33
Lower limb	1	1.67
Trunk	1	1.67
Multiple sites	2	3.33
Type of lesion		
Nodules	38	63.33
Papules	4	6.67
Plaques	13	21.67
Abscesses	20	33.33
Ulcers	3	5
No. of sites		
Single	13	21.67
Multiple	47	78.33
Pattern of distribution		
Lymphocutaneous	23	45.8
Non-lymphocutaneous	29	37.5
Deep infection	4	8.3
Disseminated cutaneous	4	8.3

**Figure 1:**
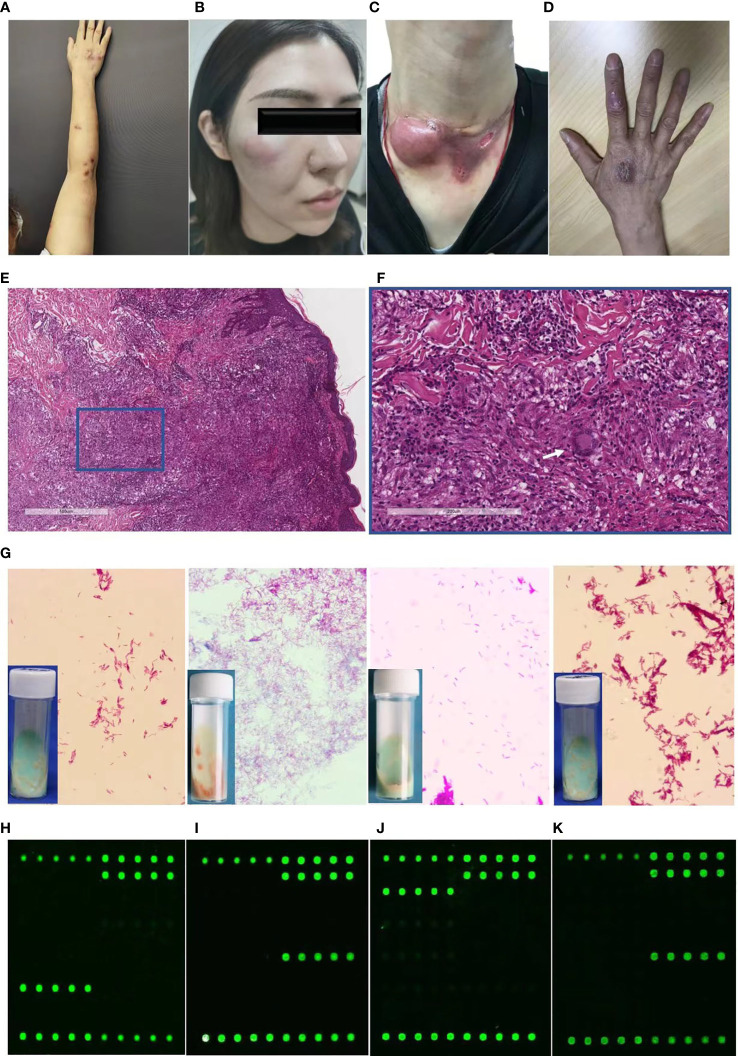
Diagnostic profile of clinical images, histopathological findings, Ziehl-Neelsen staining, and DNA microarray chip assay of participants with cutaneous mycobacterial infections. **(A-C)** Skin lesions of participants with cutaneous infection of *M. marinum* **(A)**, *M. abscessus* **(B)**, MTB **(C)**, and *M. chelonae* **(D)**. **(E, F)** Hematoxylin and eosin staining revealing acanthosis, pseudoepitheliomatous hyperplasia or exocytosis, diffuse inflammatory cells infiltration in the dermis, including multinucleated giant cells (white arrow), lymphocytes, neutrophils, and plasmocytes (magnification: A, 40×; B, 200×). **(G)** Ziehl-Neelsen staining of isolates from skin tissue culture showing red club-shaped filaments. **(H-K)** Mapped images of the DNA microarray chip assay of skin tissue confirming infection with *M. marinum* **(H)**, *M. abscessus* **(I)**, MTB **(J)**, and *M. chelonae* **(K)**.

The skin lesions were initially localized on the hand (55.00%) followed by the face (30.00%). These predilection sites were mainly located on vulnerability or injection sites. The following skin presentations were documented: nodules (63.33%, **Figure 1A**), abscesses (33.33%, **Figure 1B**), ulcers (5.00%, **Figure 1C**), and papules and plaques (6.67% and 21.67%, respectively, **Figure 1D**). Of the participants, 13 (21.67%) had single lesions, while 47 (78.33%) had multiple lesions. Lymphocutaneous lesions were present in 23 participants (45.8%), of whom 22 were infected with *M. marinum* and one with *M. chelonae*. Non-lymphocutaneous lesions were seen in 29 participants (37.5%), of whom 12 were infected with *M. marinum*, 15 with *M. abscessus*, one case each with MTB and *M. chelonae*. Deep infection was seen in four participants, two of whom were infected with MTB and *M. abscessus*. Disseminated cutaneous lesions were present in 4 participants, of whom 2 cases were infected with MTB and one each with *M. marinum* and *M. abscessus*.

### 3.3 Diverse manifestation of laboratory findings in participants with cutaneous mycobacterial infections

Histopathological examination of the specimens revealed epidermal changes in the form of acanthosis, pseudoepitheliomatous hyperplasia, or exocytosis (**Figure 1E**). Diffuse inflammatory cell infiltration was seen in the dermis comprising multinucleated giant cells, lymphocytes, neutrophils, and plasmocytes (**Figure 1F**), which was manifested in the form of an infectious granuloma (**Figures 1E, F**). The Ziehl-Neelsen staining and Periodic-acid-Schiff were negative in all the analyzed skin biopsy specimens.

Identification of pathogens from skin tissues subjected to mycobacterial culture for 4-12 weeks revealed distinct growth rate for diverse mycobacterial species. Yellowish-white wet colonies were observed in the Lowenstein-Jensen media cultures, while all isolates showed red club-shaped filaments in Ziehl-Neelsen staining (**Figure 1G**).

For the DNA microarray chip assay, the mapped images of MTB and 16 NTM were as described previously, with each of the 17 mycobacteria having their own graphic features (Zhu et al., 2010). Analysis of the images from the present study that were directly detected in the skin tissue compared to that of the mapped images revealed the presence of pathogenic mycobacterial species, such as *M. marinum* (**Figure 1H**), *M. abscessus* (**Figure 1I**), MTB (**Figure 1J**), and *M. chelonae* (**Figure 1K**).

### 3.4 Enhanced diagnostic sensitivity of DNA microarray chip assay over the skin tissue culture method in detecting cutaneous mycobacterial infections

According to the defined diagnostic criteria for cutaneous mycobacterial infections, 42 cases (70%) were diagnosed positive with the skin tissue culture method and 55 cases (91.67%) using the DNA microarray chip assay, while both assays together detected 56 (93.33%) positive cases. Among the 56 cases, 41 were positive for both the culture and DNA microarray chip assay, 14 were positive for DNA microarray chip assay but negative for culture, and one case was positive for culture but negative for DNA microarray chip assay. Four cases were negative with both culture and DNA microarray chip assay but were confirmed as positive by macrogenomic detection of pathogenic microorganisms.

The reference standard of cutaneous mycobacterial infection calculated based on the defined diagnostic criteria indicated the sensitivity, specificity and accuracy of the skin tissue culture to be 70.00% (95% CI: 56.63-80.80%), 100% (95% CI: 77.08-100%), 76.62% (67.17-86.07%), respectively, and that of the DNA microarray chip assay as 91.67% (95% CI: 80.88-96.89%), 100% (95% CI: 77.08-100%), and 93.51% (95% CI: 88.01-99.01%), respectively (**Table 3**). The sensitivity and accuracy of DNA microarray chip assay were significantly higher than those of the culture method (*P* = 0.003 and *P*= 0.003, respectively). The positive likelihood ratio of both the skin tissue culture method and DNA microarray chip assay was >10. The negative likelihood ratio of the skin tissue culture method was 30.00% (95% CI: 20.38-44.16%) and that of the DNA microarray chip assay was 8.33% (95% CI: 3.60-19.29%), indicating a statistically significant difference (*P* = 0.032). The Youden’s index of skin tissue culture method was significantly lower (70.00%, 95% CI: 56.63-80.80%) than that of the DNA microarray chip assay (91.67%, 95%CI: 80.88-96.89%) (*P* = 0.003) (**Table 4**).

**Table 3:** Sensitivities, specificity and accuracy of the skin culture method and DNA microarray chip assay in the diagnosis of cutaneous mycobacterial infections.

		Gold standard					
		Positive ^a^ (n = 60)	Negative ^b^ (n = 17)	Total (n = 77)	Sensitivity ^c^ (95% CI)	Specificity (95% CI)	Accuracy ^d^ (95% CI)
**Skin tissue culture**	Positive	42	0	42	70.00% (56.63-80.80%)	100% (77.08-100%)	76.62% (67.17-86.07%)
	Negative	18	17	35			
**DNA microarray chip**	Positive	55	0	55	91.67% (80.88-96.89%)	100% (77.08-100%)	93.51% (88.01-99.01%)
	Negative	5	17	22			

a Positive consists of 60 patients with cutanrous mycobacterial infections, who met diagnostic criteria in **2.4**.

b Negative consists of 17 skin samples from 8 patients who were diagnosed with deep dermatophytosis, 5 with pigmented nevus, 2 with epidermoid cysts and 2 with seborrheic keratosis.

c Comparison of sensitivity between the skin tissue culture and DNA microarray chip assay in the diagnosis of cutaneous mycobacterial infections, P = 0.003.

d Comparison of accuracy between the skin tissue culture and DNA microarray chip assay in diagnosis of cutaneous mycobacterial infection, P = 0.003.

**Table 4:** PLR, NLR, Youden’s index and OR of the skin tissue culture method and DNA microarray chip assay.

	PLR (95% CI)	NLR ^a^ (95% CI)	Youden’s index ^b^ (%)	OR
**Skin tissue culture**	>10	30.00% (20.38-44.16%)	70.00% (56.63-80.80%)	>1
**DNA microarray chip**	>10	8.33% (3.60-19.29%)	91.67% (80.88-96.89%)	>1

PLR, positive likelihood ratio; NLR, negative likelihood ratio; OR, odds ratio.

a Comparison of NLR between the skin tissue culture method and DNA microarray chip assay in the diagnosis of cutaneous mycobacterial infections, P = 0.032.

b Comparison of OR between the skin tissue culture method and DNA microarray chip assay in the diagnosis of cutaneous mycobacterial infections, P = 0.003.

### 3.5 Antibiotic use associated with false-negative results in the skin tissue culture method based diagnosis of cutaneous mycobacterial infections

Among the 60 cases of cutaneous mycobacterial infection confirmed, 42 were identified as positive by the skin tissue culture method, while 18 were falsely negative. Logistic regression analysis was performed to identify factors associated with the occurrence of false negative results in the skin tissue culture method. The results revealed a significant association with antibiotic usage, but no significant associations were observed with gender, age, duration, or pattern of distribution of skin lesions. Participants with a history of topical or systemic antibiotic use had an 11.81-fold (95% CI: 1.71-81.59) and 36.22-fold (95% CI: 3.63-361.09) increased risk of false negative results, respectively, compared to those with no history of antibiotic use in the skin tissue culture method (**Table 5**).

**Table 5:** Correlated factors for missed diagnosis of cutaneous mycobacterial infection in the skin tissue culture method.

	Positive rate	Skin tissue culture	
		OR (95% CI)	*P*-value
Sex			
Male	13 (61.25%)	Reference	
Female	29 (65.91%)	0.36 (0.06-2.36)	0.288
Age			
0~39 years	15 (65.22%)	Reference	
40~59 years	15 (71.43%)	1.22 (0.15-9.56)	0.854
≥60 years	12 (75.00%)	0.69 (0.10-4.74)	0.709
Duration, mo.			
≤3	22 (84.62%)	Reference	
>3,≤6	11 (74.33%)	3.23(0.74-24.29)	0.106
>6	9 (47.37%)	2.04(0.30-13.90)	0.469
History of antibiotic usage			
None	22 (95.65%)	Reference	
Topical antibiotic	11 (73.33%)	*11.81 (1.71-81.59)*	*0.012*
Systemic antibiotic	9 (40.91%)	*36.22(3.63-361.09)*	*0.002*
Pattern of distribution			
Lesion restricted to primary sites	21 (72.41%)	Reference	
Lesions progressed to distant sites	21 (67.74%)	2.27(0.40-12.94)	0.356

OR, odds ratio.

## 4 Discussion

The genus mycobacterium is the only entity within the Mycobacteriaceae family (order Actinomycetales, phylum Actinobacteria) that consists of a distinct group of aerobic, nonmotile, nonspore-forming, Gram-positive bacilli (Grange, 1996). Lack of efficient diagnostic methods overlook the detection of Mycobacterium as causative agents of cutaneous and subcutaneous infection in clinical practice (Peters et al., 2016). The present study identified 5 MTB and 55 NTM positive cutaneous infections by combining clinical spectrum and etiological examination. In general, the incidence of NTM infections has been on the rise (Wentworth et al., 2013; Mei et al., 2019; Yeo et al., 2019) and the same was evidenced by the increased number of cutaneous mycobacterial infection cases detected from 2019 to 2021 in our hospital. In addition to the general increase of infections, improved diagnostic accuracy was responsible for the augmented incidence of cutaneous mycobacterial infections (Wang and Pancholi, 2014). The present three-year study found that *M. marinum* infections were predominant over other mycobacterial species in causing cutaneous mycobacterial infections, a trend that has been previously reported (Mei et al., 2019). The surge of *M. abscessus* infection among young women, with the emergence of diverse unscheduled cosmetic infections, made it the second most causative pathogen of cutaneous mycobacterial infections (Eustace et al., 2016, Yang et al., 2017).

Cutaneous mycobacterial infection usually follows inoculation through contact of damaged skin with environmental niches such as tap water or through invasive medical methods (Bartralot et al., 2005). Infection with *M. marinum* was the most frequent cause of aquatic trauma localized on the hand and forearm, while infection with *M. abscessus* mostly occurred after cosmetic procedures and was restricted to the face. Clinical manifestation and incubation period of these infections were heterogeneous depending on the modality of mycobacterial acquisition, bacterial load, virulence, and host immune status. While cutaneous *M. marinum* infection was manifested as an isolated lesion at the site of trauma or in the form of lymphocutaneous localized infections, *M. abscessus* infections were seen in the form of multiple abscesses upon onset at cosmetic injection sites. In the current study, among the detected 5 cases of MTB infections, 2 were of lupus vulgaris, 2 of scrofulphyma, and a case was of scrofuloderma. Of the two *M. chelonae* infections, a case was similar to *M. marinum* infection presenting lymphocutaneous pattern after aquatic trauma, while the other resembled *M. abscessus* infection and appeared at sites of latrogenic injection. Thus, clinical manifestations of cutaneous mycobacterial infections lack significant pathognomonic characteristics, leaving the diagnosis to be based largely on etiological examination. Cutaneous mycobacterial infections are frequently underdiagnosed because of the limited laboratory facilities and examinations. The traditional detection techniques (Kromer et al., 2019) mostly include histopathology, skin tissue culture, and PCR-bases methods (Abdalla et al., 2009). Histopathologic analyses are nonspecific usually manifesting in the form of granulomatous inflammatory cell infiltrates that are difficult to differentiate from syphilis, granulomatous rosacea, skin tumor (Teh et al., 2013), cutaneous leishmaniasis (Vanlier et al., 2021) and deep dermatophytosis. Thus, histopathologic diagnosis is generally combined with Ziehl-Neelsen staining, particularly for lesions that have a high bacterial load. Although positive Ziehl-Neelsen staining supports the diagnosis of cutaneous mycobacterial infection, it fails to identify the pathogenic bacterial species based on morphological characteristics. Identification of clinical mycobacterium isolates at the species level is pivotal for the formulation of antibiotic treatment regimens. Presently, skin tissue culture is considered the sole reliable method for determining and identifying the presence of mycobacterial species and their respective sensitivity to drugs (Samper and González-Martin, 2018). Diverse mycobacterial species exhibit distinct culture conditions with specific incubation temperatures and time (Wallace et al., 2021). Majority of the mycobacterium grow at 35-37°C. However, *M. ulcerans* and *M. marinum* grow best at 30-32°C, while *M. kansasii* grows at 31°C and 37°C with specific growth characteristics exhibited subsequent to light exposure. Furthermore, recovery of all mycobacterial species requires culturing for several weeks. Incubation period for cultures of *M. marinum*, MTB (Handog et al., 2008), and *M. ulcerans* are 2-4, 2-6, and 6-12 weeks, respectively. Thus, the overall operational process of the skin tissue culture method is time-consuming and labor-intensive. In addition, the participants have to endure long waiting period for results with probable aggravation of skin lesions during the interval. Hence, availability of rapid and simple molecular techniques for the diagnosis of cutaneous mycobacterial infections is the need of the hour.

The advent of molecular biology techniques that led to significant advances in DNA amplification and hybridization techniques has significantly aided the rectification of current flaws in the diagnosis of cutaneous mycobacterial infections (Jagielski et al., 2016). DNA sequencing, PCR-restriction fragment length polymorphism (RFLP) assays (Wong et al., 2003), gene chip assays (Chang et al., 2010), and commercial kits, such as GenoType and INNO-LiPA, are established nucleic acid-based assays for identification of mycobacterial infections (Zhu et al., 2010). DNA sequencing, especially metagenomic next-generation sequencing (mNGS) (Kong et al., 2022), has greater efficiency, sensitivity, and specificity in detection of pathogenic bacteria, and can even detect rare pathogens. However, the detection of non-specific pathogens by mNGS is possible, creating difficulties in differentiating between contaminants, colonizers, and pathogens; furthermore, mNGS is not cheap and could be prohibitively expensive for patients. A positive PCR-RFLP result (Wong et al., 2003) will only confirm mycobacterial infection, without identification of the pathogenic mycobacterial species. Previously used gene chip assays (Chang et al., 2010) and DNA probes (Chung et al., 2018) have limited detection ranges, and are also not available for most NTM species; moreover, these methods can only be used for detection in specific samples types, such as mycobacterial isolates, sputum, pus. Both the GenoType and the INNO-LiPA assay are line-probe assays and, because of the space limitations of the strip, it is difficult to include more probes for the identification of a greater variety of species in a single experiment. Since the molecular methods mentioned above have flawed including high price, low sensitivity, restricted detection range, and low efficiency (Yüksel and Tansel, 2009; Röltgen et al., 2012), it makes meeting the clinical diagnostic demand challenging.

The newly designed DNA microarray biochip system with species-specific 16S rRNA sequences for identifying 17 mycobacterial species could be promising for diagnosing mycobacterial infections (Zhu et al., 2010). Compared to other currently used diagnostic methods, the advantages of DNA microarray biochip-based diagnostic method are as follows: (i) It have simplified the procedure for nucleic acid extraction, convenient operation process, and concise interpretation of the result. The time span from the start of the DNA microarray chip assay to the acquisition of the results averages 6 h. Compared to traditional culture methods that can take 2-12 weeks, the use of the DNA microarray biochip greatly shortens the diagnostic procedure. (ii) Each DNA microarray chip can simultaneously and rapidly test 4 samples, while each experiment can test 24 chips simultaneously. This high-throughput feature reduces the need for repetitive work by the technician and can thus meet busy clinical requirements. (iii) The cost of mycobacterial detection by the DNA microarray biochip is about $30 per sample, which is much cheaper than that of other molecular diagnostic methods, especially mNGS. The low cost is more acceptable to patients and more conducive to clinical application. Thus, the DNA microarray biochip assay is a simple, rapid, high-throughput, and economical diagnostic detection method that is ideal for the clinical screening of large numbers of samples.

The DNA microarray chip technology had been widely used for the diagnosis of pulmonary mycobacterial infections employing sputum specimens (Shi et al., 2012). Skin represents the most common extrapulmonary organ involved in mycobacterial infections (Rajendran et al., 2021) and therefore warrants an urgent need for efficient and accurate diagnostic methods. In the present study, a novel DNA microarray chip was used for the detection of mycobacterial isolates in clinical skin tissue specimens and was found to be more sensitive and accurate compared to that of the conventional culture method (70.00% vs 91.67%; 77.92% vs 93.51%). The positive likelihood ratios for both DNA microarray chip and culture method were more than 10, which provided clear evidence that they were significantly more likely to diagnose cutaneous mycobacterial infection accurately. The negative likelihood ratio was significantly smaller for DNA microarray chip compared with the culture method, suggesting that the negative results of DNA microarray chip were more likely to true negative. The Youden’s index for DNA microarray chip was superior to that for culture method, indicating that the results of DNA microarray chip were more reliable. Diagnostic detection sensitivity of the skin tissue culture method was susceptible to antibiotic usage, since 63.33% of the participants with cutaneous mycobacterial infection had a history of systemic or topical antibiotic abuse before he/she visited the hospital. Inappropriate use of antibiotics might have reduced mycobacterial infection to a certain extent lessening the mycobacterial load or activity and could have contributed to the false negative results in the skin tissue culture method. However, diagnosis of cutaneous mycobacterial infection by the DNA microarray chip assay was not influenced by antibiotic treatment, probably due to the detection of DNA sequences from bacterial debris or dead bacteria. Thus, the DNA microarray chip assay is a highly sensitive and reliable clinical diagnostic technique for the early etiological diagnosis of cutaneous mycobacterial infection that could facilitate timely intervention of pathogen-adapted antimicrobial therapy.

The results of the present study indicate DNA microarray chip assay as an effective method for the early diagnosis of cutaneous mycobacterial infections. However, the method is limited in differentiating *M. marinum*-*M. ulcerans* and *M. chelonae*-*M. abscessus* paired infections, as each of these paired NTMS have the same 16S rRNA sequences. Thus, the method is reliable as a diagnostic tool in combination with medical history and clinical symptoms. This limitation could potentially be overcome by using combined probes 16S rRNA and 23S rRNA, or 5S rRNA genes. Further, the DNA microarray chip assay is limited in providing information related to drug-resistance and is therefore inefficient in completely replacing the skin tissue culture method, which still remain an essential part of the diagnosis and treatment protocol. Future research is required to develop modified DNA chip related technologies for the detection of drug resistance in the diagnosis of cutaneous mycobacterial infections.

## 5 Conclusion

The present study reports on the incidence, clinical characteristics, and pathogenic profile of mycobacteria associated with cutaneous mycobacterial infections in Shanghai, China. Achieving accurate and rapid diagnosis of cutaneous mycobacterial infections remains a major clinical challenge that requires specific and timely intervention for effective treatment and improved prognosis. The DNA microarray chip assay evaluated in this study provides a simple, rapid, high-throughput, and reliable method for identifying 17 mycobacterial species, and overcomes the limitations associated with traditional detection methods for diagnosing cutaneous mycobacterial infections. Therefore, the DNA microarray chip assay holds great potential as a clinical tool for diagnosing cutaneous mycobacterial infections.

## Data availability statement

The datasets presented in this study can be found in online repositories. The names of the repository/repositories and accession number(s) can be found below: https://www.ncbi.nlm.nih.gov/, OQ594967 OQ594968 OQ594969 OQ594970 OQ594971 OQ594972 OQ594973 OQ594974 OQ594975 OQ594976 OQ594977 OQ594978 OQ594979 OQ594980 OQ594981 OQ594982 OQ594983 OQ594984 OQ594985 OQ594986 OQ594987 OQ594988 OQ594989 OQ594990 OQ594991 OQ594992 OQ594993 OQ594994 OQ594995 OQ594996 OQ594997 OQ594998 OQ594999 OQ595000 OQ595001 OQ595002 OQ595003 OQ595004 OQ595005 OQ595006 OQ595007 OQ595008.

## Ethics statement

Written informed consent was obtained from the individual(s) for the publication of any potentially identifiable images or data included in this article.

## Author contributions

QY and YW designed and drafted the work, contributed to the DNA microarray chip testing, analysis, acquisition, and interpretation of data; ZG, HY, and SL performed the laboratory tests and analyzed the data; JT reviewed the cases and provided clinical information; LY designed the study, revised, and finalized the manuscript. All authors contributed to the article and approved the submitted version.
