# Supplementary material for: DNA microarray chip assay in new use: early diagnostic value in cutaneous mycobacterial infection
Source: Front Cell Infect Microbiol. 2023 Jul 1;13:1183078. doi: 10.3389/fcimb.2023.1183078 (PMC10349391; doi:10.3389/fcimb.2023.1183078)
Supplement: Supplementary file 1 [file DataSheet_1.docx]

Supplementary Material

# Supplementary Figure 1


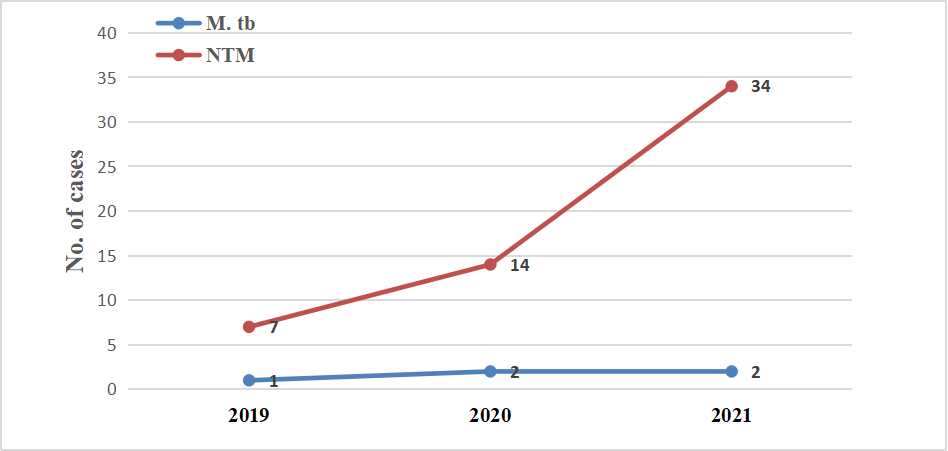


**Supplementary Figure 1.** Cases of cutaneous mycobacterial infection in Shanghai Dermatology Hospital (2019-2021).
